# A prospective evaluation of a largely cementless total knee arthroplasty cohort without patellar resurfacing: 10-year outcomes and survivorship

**DOI:** 10.1186/s12891-018-2128-1

**Published:** 2018-06-26

**Authors:** Richard J. Napier, Christopher O’Neill, Seamus O’Brien, Emer Doran, Brian Mockford, Jens Boldt, David E. Beverland

**Affiliations:** 1 Orthopaedic Outcomes Assessment Unit, Musgrave Park Hospital, Stockman’s Lane, Belfast, BT9 7JB Northern Ireland; 2 Akutklinik Siloah, Worbstrasse 324, CH 3073 Guemligen, Switzerland

**Keywords:** Cementless TKA, LCS total knee, Cementless knee survivorship, Knee replacement

## Abstract

**Background:**

The theoretical benefits of a mobile bearing design in Total Knee Arthroplasty (TKA) include increased articular surface conformity with a reduction in both polyethylene wear and implant interface shear. However, to date these theoretical advantages have not been translated into published evidence of superior survivorship. This paper presents the results of a prospective, non-comparative study evaluating the performance of the mobile bearing Low Contact Stress LCS Complete Rotating Platform TKA in a largely cementless cohort without patellar resurfacing.

**Methods:**

237 consecutive patients (240 knees) undergoing primary TKA were prospectively recruited. All received the LCS Complete Rotating Platform TKA (DePuy International, Leeds, UK). Clinical and radiographic assessments were performed at 3, 12, 60 and 120 months post-operatively. Radiographic evaluation was performed by an independent external surgeon.

**Results:**

The mean age was 70.3 years. 77.5% of cases were cementless. Radiographic assessment suggested excellent femoral component fixation. 22 tibial radiolucent lines (RLLs) > 1 mm were observed in 12 knees. No RLLs were progressive. There have been two revisions; one for late infection and one for aseptic loosening. No patients underwent secondary patellar resurfacing. The cumulative implant survivorship, using component revision for any reason as the endpoint, was 98.9% (95% CI, 95.6 to 99.7%) at 10 years.

**Conclusions:**

The excellent survivorship at a minimum 10-year follow-up supports the use of uncemented porous coated fixation without patellar resurfacing with the non-posterior stabilized LCS Complete Rotating Platform TKA.

## Background

The theoretical benefits of a rotating platform design in Total Knee Arthroplasty (TKA) are well-documented [1, 2]. The concept of increasing the congruity of the articular surface to reduce polyethylene wear rates, coupled with the use of a mobile bearing (MB) to minimize constraint forces associated with mechanical loosening should theoretically be associated with greater long-term survivorship [3]. However to date such advantages have not been demonstrated in the literature [4–9].

Although cemented fixation for TKA is supported within the literature as offering excellent long-term survivorship [10], cementless fixation has many benefits including shorter surgical time, which may reduce infection rates [11], preservation of bone stock for revision procedures [12], and prevention of third body cement wear. The most common cause of late failure in TKA remains aseptic loosening particularly the tibial component [13–16]. A recent Cochrane review suggested if good early fixation is achieved the potential for later aseptic loosening is reduced by up to half compared to cemented TKA [17].

Patellar resurfacing also creates significant debate among knee surgeons with numerous studies both for [18–21] and against [22, 23]. Due to the wide variety of trochlear geometry between TKA designs, it may be more appropriate to refer to specific designs when debating patellar resurfacing [24–26]. Additional factors such as surgical technique and the primary indication for knee arthroplasty also contribute to reducing anterior knee pain post-operatively [27, 28]. This prospective, non-comparative study aimed to evaluating the performance of a rotating platform TKA using predominantly cementless fixation and without patellar resurfacing at a minimum 10-year follow-up.

## Methods

Between March 2002–January 2003, 237 consecutive patients (240 knees) scheduled for primary TKA at a regional orthopaedic centre were prospectively recruited. Exclusion criteria included: previous knee surgery (except for open or arthroscopic meniscectomy) and/or an inability to give informed consent. Institutional Review Board (IRB) approval was obtained from the regional research and ethics committee. (Study reference ORECNI-335-01) and written consent was obtained from all patients.

**Fig. 1:**
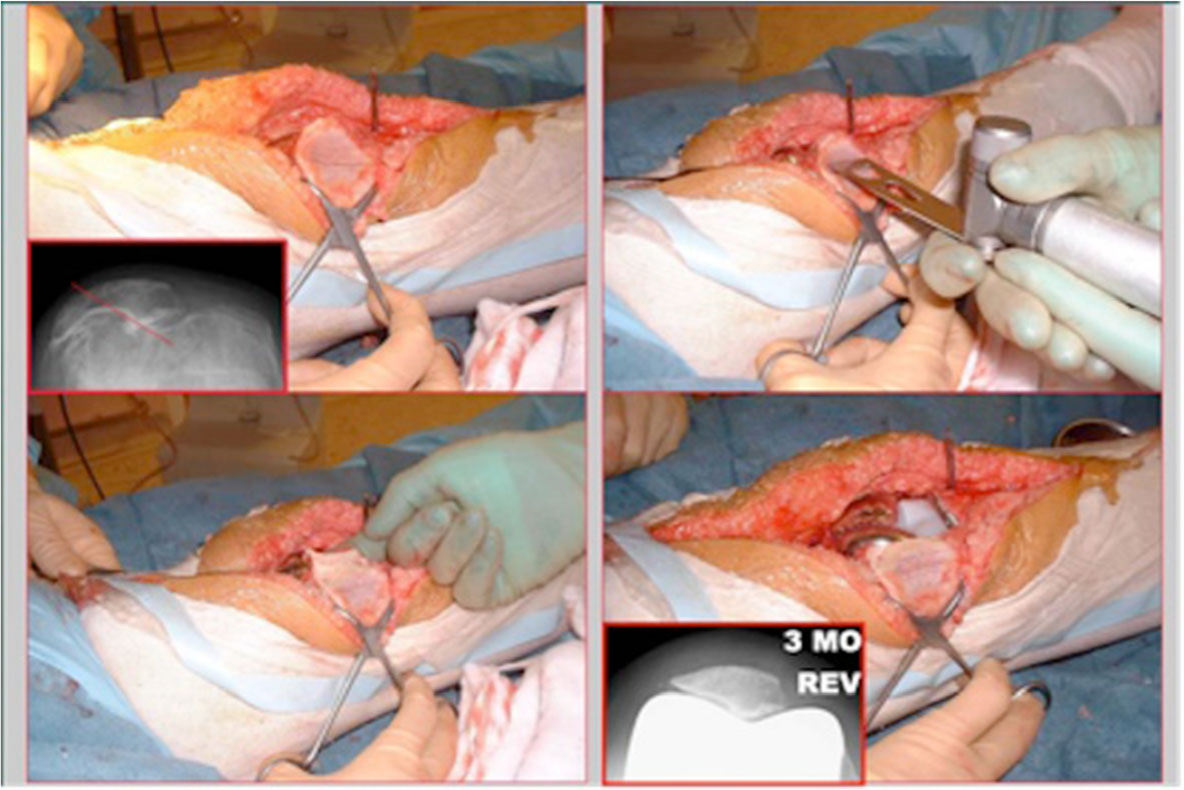
Demonstrating the technique for patellar contouring with an inset showing preoperative and three-month skyline views

All patients received a LCS Complete Rotating Platform TKA (DePuy International, Leeds, UK), without patellar resurfacing. Surgery was performed under the care of the senior author. The philosophy for alignment was to achieve a balanced knee through bone cuts rather than soft tissue releases, and not to aim for a neutral mechanical axis [29, 30]. At the beginning of the study hybrid fixation with a cemented tibia was used in female patients with valgus deformities due to concern regarding early post-operative tibial insufficiency fractures [31]. Prophylactic antibiotics were administered prior to tourniquet inflation, with the tourniquet being released after deep closure. A medial Insall approach was utilized in all cases. The patella underwent removal of rim osteophytes, with a lateral patellar release only performed in cases were persistent patellar tilt or a mal-tracking was observed. In more severe cases (Sperner grade IV) a lateral facetectomy was performed [Fig. 1] [32]. No patellae underwent resurfacing. Thirty knees underwent lateral release and 1 required facetectomy.

All knees were closed in flexion and kept flexed on a pillow for 6 h to reduce blood loss [33]. Patients without a personal history of venous thromboembolism received oral aspirin 150 mg once daily for 6 weeks as thromboprophylaxis [34, 35]. Anesthesia consisted of a spinal anesthetic combined with regional femoral and sciatic nerve blocks. All patients were mobilized full weight-bearing on the first post-operative day.

Patients were reviewed by Arthroplasty Care Practitioners [36] with clinical and radiographic assessments at 3, 12, 60 and 120 months post-operatively.

Clinical outcome was assessed by comparison of pre and postoperative Oxford Knee Scores (OKS), American Knee Society Scores (AKSS) and Bartlett Patellar Scores (BPS). Quality of life was assessed using the 12 item Short Form Health Survey (SF-12).

**Fig. 2:**
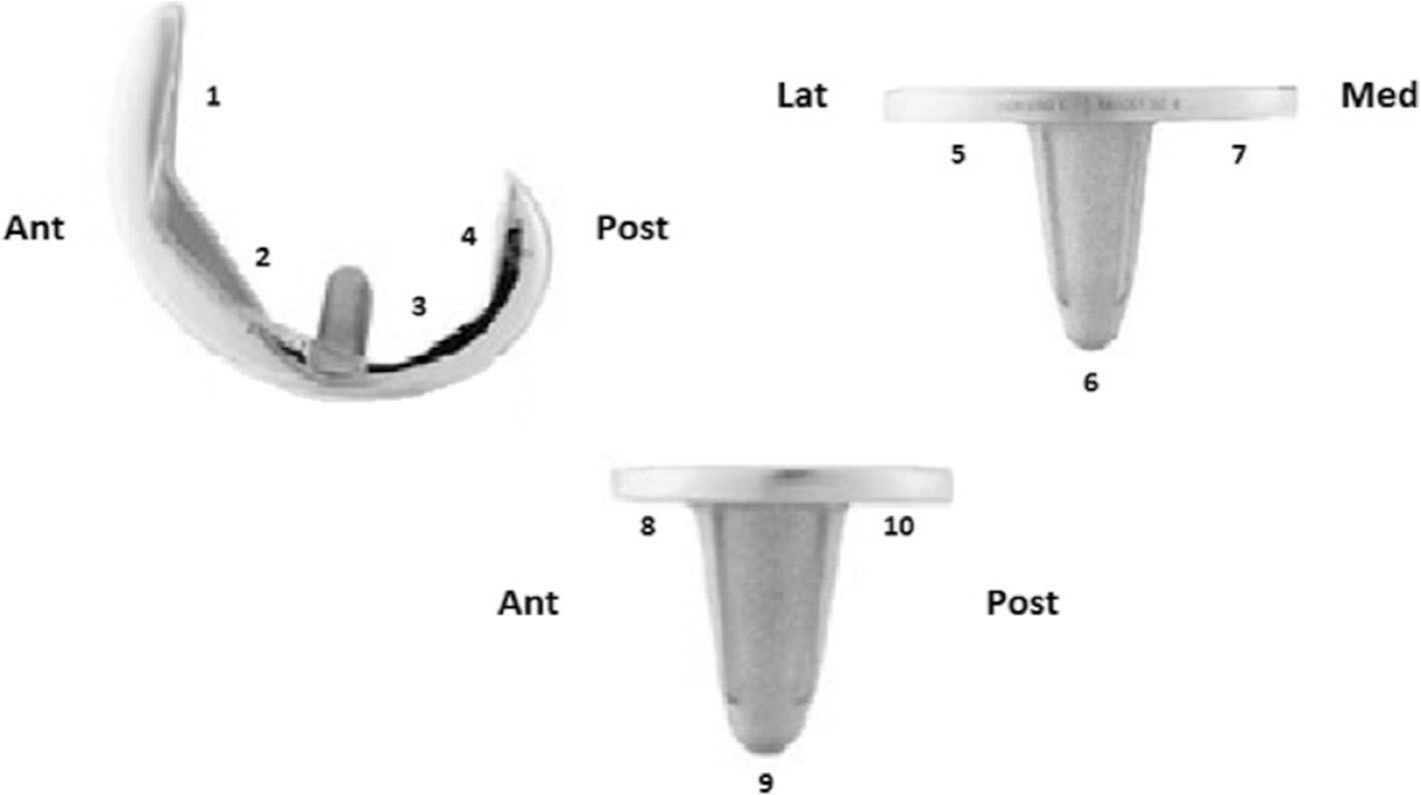
Modified scoring system with femoral (1–4) and tibial (5–10) zones

Radiographic evaluation was performed by an independent external surgeon. Standardised erect antero-posterior (AP), lateral and skyline patellar views were obtained. Radiographs were assessed for the presence of radiolucent lines (RLLs) and osteolytic defects. A RLL was defined as a radiolucency of ≥1 mm in greater than 50% of a zone. The zones are illustrated in Fig. 2. Revision for any reason was used as the end point for survivorship. Of the 237 patients enrolled in the study, 84 subjects were withdrawn at the end of 10 years: 70 Deceased, 2 Revisions, 12 lost to follow-up (7 due to ill-health, 3 moved away and 2 untraceable).

### Statistical analysis

Survivorship was determined using the Kaplan-Meier method. Subjects who died or were lost to follow-up were included in the survivorship analysis and in the calculation of the 95% confidence intervals.

## Results

The mean age of the patients at the time of surgery was 70.3 years (range 39–89). The series included 91 males and 146 females. One female and two males had bilateral staged knee replacements. Osteoarthritis was the indication for surgery in 94% of cases (226 knees). Other indications included inflammatory arthropathy 4.6% (11 knees) and post-traumatic arthritis 0.4% (1 knee). Four knees within this study underwent TKA for isolated patellofemoral disease [28]. Patient demographics are summarised in Table 1.

**Table 1:** Demographics of Enrolled Patients

Patients/Knee (n)	237//240
Gender (Patients (%)	
Male	91 (38.4%)
Female	146 (61.6%)
Age at operations (years)	
Mean (years)	70.3
Minimum (years)	39
Maximum (years)	89
Age groups (knees) (%)	
< 50 years	7 (3%)
50–69 years	94 (39%)
70–79 years	110 (46%)
80–89 years	29 (12%)
Body Mass Index (kg/m^2^)	
Mean	28.8
Minimum	18
Maximum	45
BMI groups (kg/m^2^)	
Underweight (< 18.5)	1 (0.5%)
Normal (18.5–24.9)	45 (19%)
Overweight (25.0–29.9)	103 (43%)
Obese class 1(30.0–34.9)	71 (29.5%)
Obese class 2 (35–39.9)	17 (7%)
Morbidly obese ≥40	3 (1%)
Primary Diagnosis	
Osteoarthritis	226 (94.2%)
Post traumatic Arthritis	1 (0.4%)
Rheumatoid Arthritis	11 (4.6%)
Other	2 (0.8%)

Pre-operative radiological alignment was predominantly varus with 165 (68.75%) knees being in varus alignment (Mean Hip Knee Ankle angle 10.2^o^) and 75 (31.25%) knees in valgus (Mean 7.9^o^). (Range 24^o^ varus - 38^o^ valgus).

Of the 240 knees in the study 186 (77.5%) were cementless, 53 (22.0%) were hybrid fixation with a cemented tibial component, and 1 (0.4%) was an all cemented knee. 81% of the patients were overweight or obese (Mean BMI 28.8 kg/m^2^). Of the 54 cemented tibias, 53 were female and of those 49 had either a neutral or valgus preoperative alignment. A cemented component was used in the one male patient because a cementless component was unavailable.

### Baseline & 10-year outcome measures

When comparing validated outcome scores from pre-operatively to those at 10-year review, a clinically and statistically significant improvement in all outcome measures was observed. Importantly this included patellar scores and pain scores (Table 2).

**Table 2:** Summary of baseline and last review outcome measures

Outcome Measure	Mean Score at Baseline	Mean Score at Final Review (10 years)	Change from Baseline	*p*-value
Total Knee Score	20.9	91.0	67.21	< 0.0001
Knee Function Scores	37.7	64.8	24.93	< 0.0001
Patella Score	9.6	25.3	15.16	< 0.0001
Oxford Knee Score	10.8	35.7	24.55	< 0.0001
SF-12 Physical Component	26.9	36.1	8.53	< 0.0001
SF-12 Mental Component	42.4	50.0	6.98	< 0.0001
Knee Pain Sub Score	2.8	46.1	43.20	< 0.0001
Range of Movement (Active)	101.4	105.9	3.67	0.0185

(Note- Mean baseline score subtracted from mean final review score does not equal change from baseline due to the reduction in numbers secondary to loss to follow-up at 10-years)

### Radiographic analysis

Standardised post-operative AP and lateral radiographs were analysed using a modified Knee Society TKA Roentgenographic scoring system to determine the presence, width and location of any RLLs [37]. The AP radiograph was taken in approximately seven degrees of flexion to account for tibial resection slope and to ensure a proper view of the tibial-bone interface. The diagram in Fig. 2 shows the modified scoring system with femoral (1–4) and tibial (5–10) zones. Radiographic assessment showed no RLLs in femoral zones (1–4), suggesting excellent femoral component fixation.

Twenty-two tibial RLLs were observed in 12 subjects (12 knees) (see Table 3). Only one cemented tibial component had RLLs observed during the follow-up period (1/54(1.9%)). The total number of cementless tibial components with RLLs observed was 11/186 (5.9%), with RLLs being reported twice in one patient at two different time points and in different zones. The most common locations for RLLs were Zones 5 and 7 of the proximal tibia. No RLLs were progressive.

**Table 3:** Tibial RLL Zones identified during variable review periods

Subject #	Cemented/Non-Cemented	Follow-up Interval	Tibial Radiolucencies (mm)					
			Zone 5	Zone 6	Zone 7	Zone 8	Zone 9	Zone 10
1–026	Non-cemented	5 years	4					
1–031	Non-cemented	3 months			1			
1–053	Non-cemented	5 years					1	
1–065	Non-cemented	12 months			1	1		
1–075	Non-cemented	5 years					1	
1–157	Non-cemented	12 months	1		1	1		
1–175	Non-cemented	10 years		1				
1–186	Cemented	10 years					1	
1–215*	Non-cemented	12 months	1		1			
1–215*	Non-cemented	10 years					1	
1–228	Non-cemented	3 months	1		1			1
1–232	Non-cemented	12 months	1			1		
1–241	Non-cemented	12 months	1		1	1		

Two knees with documented RLLs at 3 months post-operatively showed full resolution at 1, 5 and 10-year review. Five knees had RLLs present for the first time at 1-year, and only 1 had RLLs visible at subsequent 5 or 10-year review and this occurred in a different location (Zone 10). Three knees had RLLs present for the first time at 5-year review, which were no longer apparent at 10-year review. Three knees had RLLs present for first time at 10-year review. There was no statistically significant difference in pain scores between cases with and without RLLs, (approximate t-test *p*-value of 0.77), suggesting neither pain nor revision was related to the presence of RLLs. Comparing RLL in cemented vs uncemented cases showed no statistical significance (Fisher’s exact *p*-value = 0.308).

### Patellofemoral alignment

Pre- and post-operative patellofemoral alignment was measured on Merchant skyline radiographs. Pre-operatively, the patella was centrally aligned in 61.9%, laterally aligned in 26.4%, and medially aligned in 11.7% of patients. At initial postoperative 3 month and final review (120 months) 99.5 and 98.9% of patellae respectively showed central patellar alignment. Patellar tilt was also evaluated on pre- and postoperative skyline radiographs. Preoperatively, 31.6% of patellae lay centrally (neutral tilt), 48.0% had lateral tilt and 20.4% had medial tilt. At the 3-month post-operative review 98.0% had neutral tilt. At 10-year follow-up, all reviewed knees exhibited neutral patellar tilt.

In this study, patients receiving cemented and uncemented tibial components were unmatched cohorts. There was no significant difference in Knee Society Pain Subscore or Knee Society Function Score between cemented and non-cemented knees at 10 years however there were fewer RLLs in the cemented tibial cohort.

**Fig. 3:**
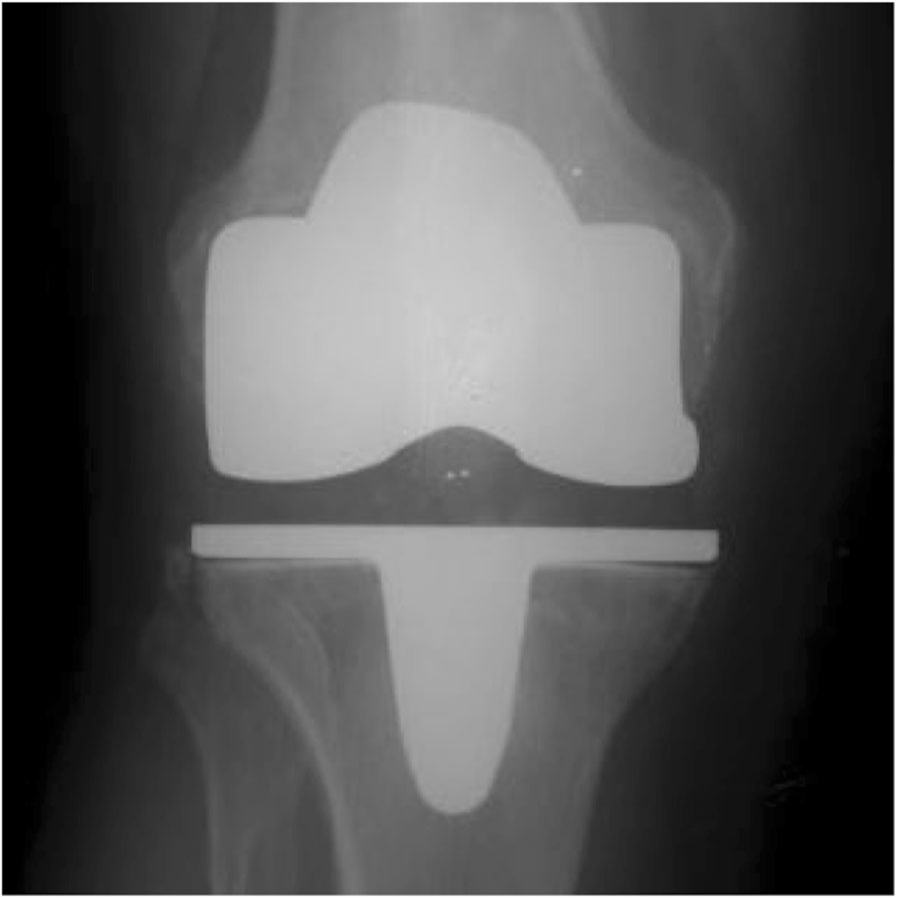
Example of RLLs in Zones 5 & 7

**Fig. 4:**
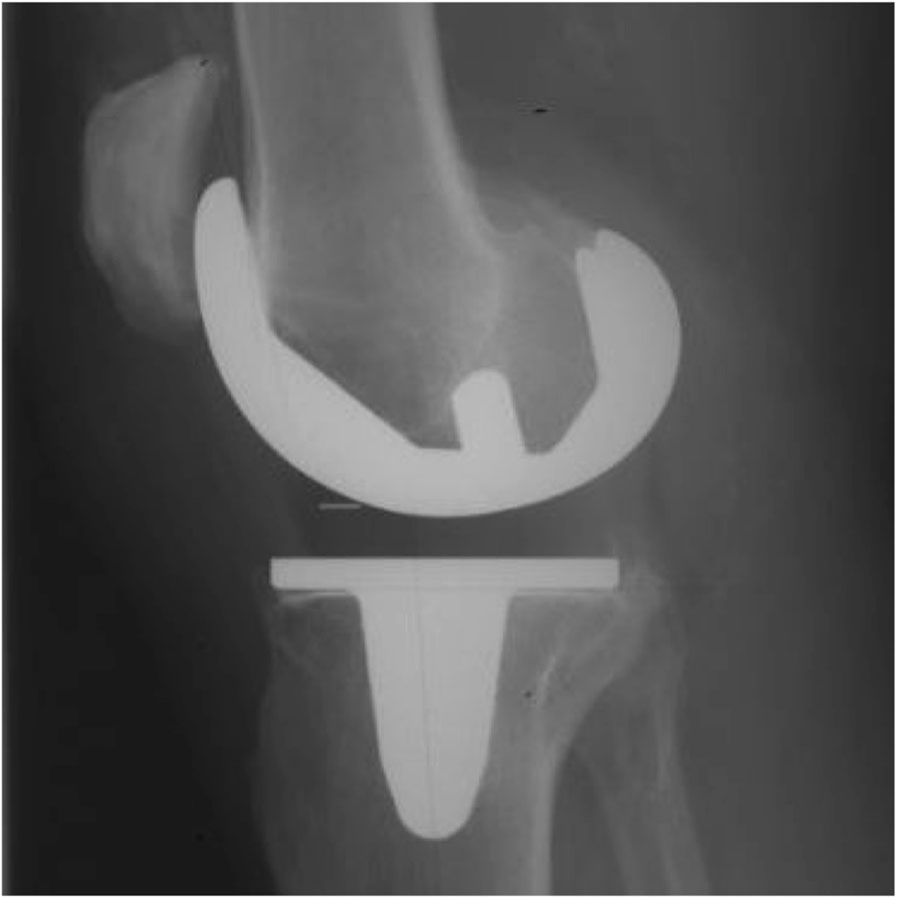
Example of RLLs in Zone 8

Of the 240 knees in the study only 2 cementless implants underwent revision. The first case was a revision for late haematogenous infection at 9.2 years post primary surgery. The second case was for aseptic loosening at 8.3 years after primary surgery. X-rays showed anterior femoral cortical scalloping with osteolysis in femoral zones 1 and 4, and tibial zones 5 and 8. Intra-operatively, the femoral component was loose and only the tibial cone remained well fixed, both components were revised. Interestingly this patient had no RLLs at the time of 5-year review and was revised prior to 10-year review (Figs. 3 and 4). Ten additional patients had further surgery. Nine were early washouts without bearing exchange (6 for infection and 3 for non-infected haematoma) and the tenth was a late (109 months) periprosthetic fracture following a road traffic collision requiring internal fixation. There were no manipulations under anaesthetic, no cases of bearing spinout and no secondary patellar resurfacings [38].

### Kaplan-Meier survivorship

**Fig. 5:**
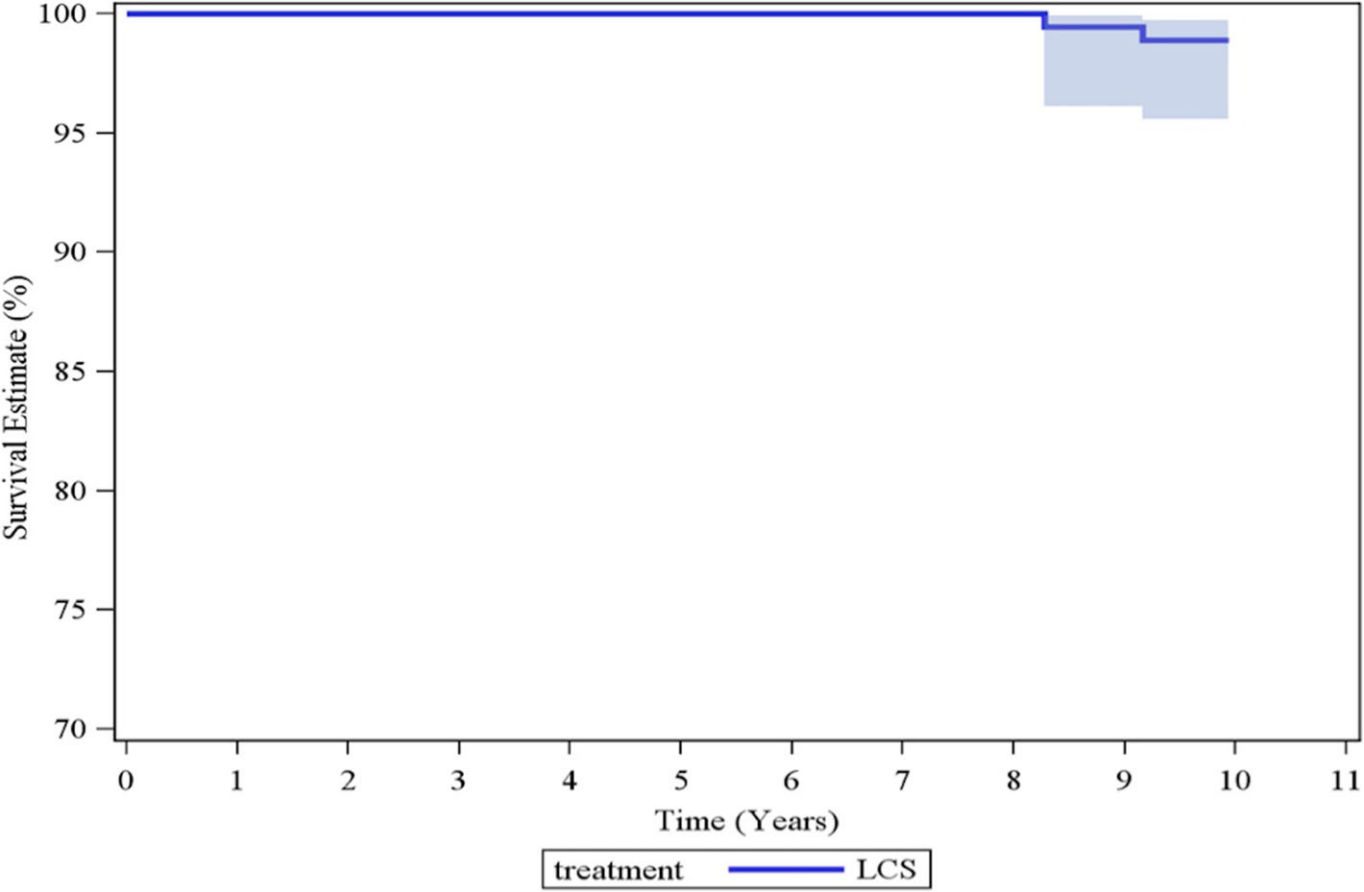
Survival estimate at 10 years in 98.9% (95% Confidence Interval: 95.6–99.7%)

The cumulative implant survivorship of the whole series using component revision for any reason as the endpoint was 98.9% (95% CI, 95.6 to 99.7%) at 10 years (Fig. 5). With revision due to infection excluded, cumulative implant survivorship increased to 99.4% (95% CI 96.1 to 99.9%) at 10 years.

## Discussion

This paper encompasses three controversial issues within TKA. Firstly, the reported cohort is largely cementless; secondly all were non-posterior stabilized mobile-bearings and thirdly the patella was never resurfaced. The survivorship of this cohort is excellent.

Cementless fixation has the benefits of bone stock preservation, prevention of third body cement wear, and shorter surgical times. [11, 12] However the registry evidence in favour of cemented as opposed to cementless TKA is compelling, with the UK, Swedish, Australian and New Zealand registries all showing superior survivorship with cemented TKA [39–42]. In the 2014 UK National Joint Registry (NJR) report [39] cementless use fell to 2.5% compared to 6.7% in 2003. Paradoxically in many areas of the world this minimal use of cementless TKA is in stark contrast to that of cementless total hip arthroplasty even though registry data also favours cement for that joint in terms of survivorship [39, 43] and the implants are more expensive.

Level I evidence in favour of cemented TKA is unconvincing. The prospective RCT by Park and Kim [44] has been used in support of cemented TKA [8]. Park and Kim’s study [44] reported on 50 patients (100 knees) who had undergone bilateral, simultaneous cruciate retaining knee replacements with one cemented and the other cementless. At a minimum follow-up of 13 years, survival of the femoral components was 100% in both groups and there was one revision for aseptic loosening of a cementless tibial component that had occurred at 1 year. No osteolysis was identified in either group. Baker et al. [45] in their prospective RCT reported no difference in the survivorship of cemented versus uncemented cruciate retaining knees at a mean follow-up of 9 years. However, cemented TKA demonstrated higher failure rates in younger, heavier men [45]. Our study population had an average BMI of 28.8, but a decade later the average BMI for patients presenting for TKA has risen to 31.8.

A meta-analysis by Gandhi et al. [10] suggested an improved survivorship of cemented as compared to uncemented implants when they looked at 15 studies selected from a total of 1292. Unfortunately, of the 15 studies only 5 were RCTs, with the other 10 being observational. Subgroup analysis of the 5 RCTs showed no survival difference. The authors noted that the patients in the cementless observational studies tended to be younger. In the absence of a clear advantage for either method of fixation the authors concluded that cement offered an economic advantage because of the reduced cost.

For both cemented and uncemented TKAs, aseptic loosening of the tibia is the most common reason for failure [14–16]. Although cement does have advantages such as augmenting bony defects or inaccurate bone cuts [46] its weakness and mode of failure is susceptibility to torsional and shear forces [47]. Posterior stabilized designs have higher stresses which hypothetically should impact on survivorship. This hypothesis is supported by results from the 2014 UK NJR [39] where cemented cruciate retaining TKAs have a better survivorship than posterior stabilized. Similarly for cementless fixed bearing (FB) TKAs these torsional and shear stresses could result in aseptic loosening if micromotion is present during early osteointegration [11]. Hypothetically if uncemented implants can survive these early threats to osteointegration by forming a biological bond between bone and prosthesis this could improve long-term survivorship. This hypothesis is supported by a Cochrane review [17], however good results have been reported even with a posterior stabilized cementless TKA [47].

In contrast to the FB TKA the MB, as well as providing low contact stress and low wear [48], also reduces the stresses applied to the implant-bone interface. [3] This should reduce wear induced osteolysis and tibial loosening however these theoretical advantages over FB TKAs have not been confirmed in the literature [4–9] even though MB TKA has shown excellent medium to long-term results in function and survivorship [49, 50].

The reduced shear stress at the implant bone interface in cementless MB TKA should facilitate early osteointegration and may contribute to the survivorship in this study. One manifestation of failure of bony ingrowth is fibrous ingrowth presenting as a progressive RLL. A recognised concern with cementless porous coated tibial trays is the appearance and interpretation of RLLs. In a patient with unexplained pain this can lead to inappropriate revision. Two types of RLL have been described, physiological or pathological [51]. Physiological lines are usually < 2 mm with a sclerotic margin, and pathological lines are thicker, with a poorly defined border, and are usually progressive [52]. In this series only 1/54 cemented tibial components exhibited RLLs compared with 12/186 of the cementless tibias. Similar lines have undoubtedly resulted in unnecessary revision. We believe lines < 1 mm represent non-pathological fibro-osseous integration which is stable and non-progressive.

Paradoxically the cemented Oxford Uni-compartmental MB knee also suffers from inappropriate revisions due to the presence of lines below the tibial tray [53], but these are infrequent with the cementless HA coated version of the tibial tray [54].

The requirement for patellar resurfacing remains contentious and contradictory [19, 21, 23]. The geometry of the different femoral components observed between knee systems may play a role in this inconsistency [19–22]. In both resurfaced and non-resurfaced patellae, contact stresses within the trochlear sulcus are dictated by the component design [24]. With posterior stabilized designs traditionally having more patellofemoral problems [55]. The LCS system exhibits a common radius of curvature similar to that found in the native knee which allows it to accommodate the unresurfaced patella favorably [56]. However, the posterior stabilized LCS Complete was removed from the Australian market following higher than expected revision when the patella was not resurfaced [57]. With the benefits of patellar resurfacing unresolved within the literature, it is important to consider the associated risks of patellar fracture, avascular necrosis, implant loosening and instability [27]. Intraoperatively, and irrespective of implant choice or decision to resurface, it is critical to achieve central patellofemoral tracking. Persistent anterior knee pain after TKA is common with and without patellar resurfacing [58, 59] and secondary resurfacing can be of questionable clinical value [18, 60].

Weaknesses of this study include the use of a number of cemented tibial components. Recruitment for this study had been on consecutive patients and the senior author’s practice at the commencement of the study was to use hybrid fixation with a cemented tibia in females with valgus deformities due to perceived concerns about tibial insufficiency fractures [31]. Since the conclusion of this study, this practice has changed, with uncemented tibial components being implanted in all cases irrespective of age, gender, or deformity. We previously reported a series of 275 consecutive valgus knees with a pre-operative deformity of ≥10° which was completed after this study. Eighty three percent were female and of those 70% were cementless. At between 5.8 and 10.5 years follow-up, there has been only one revision, which was in a cemented tibia [31]. A further limitation was patients lost to follow-up being high. There were 84 subjects who had withdrawn from the study at the end of 10-years, 83% of these were deceased.

## Conclusion

In terms of survivorship this study supports the use of non-cemented porous coated fixation without patellar resurfacing using a non-posterior stabilized mobile bearing TKA. It exhibits excellent survivorship at a minimum 10-year follow-up with the expectation that longer-term follow-up may be superior to cemented TKA. We believe lines < 1 mm represent non-pathological fibro-osseous integration which is stable and non-progressive. In the absence of definitive level-1 evidence the only clear advantage for cemented TKA is cost.

## Abbreviations

AKSS: American Knee Society Scores
AP: Antero-posterior
BPS: Bartlett Patellar Scores
FB: Fixed Bearing
IRB: Institutional Review Board
MB: Mobile bearing
NJR: National Joint Registry
OKS: Oxford knee scores
RLLs: Radiolucent lines
SF-12: 12 item Short Form Health Survey
TKA: Total Knee Arthroplasty
